# Enrichment of miR-126 enhances the effects of endothelial progenitor cell–derived microvesicles on modulating MC3T3-E1 cell function via Erk1/2-Bcl-2 signalling pathway

**DOI:** 10.1080/19336896.2019.1607464

**Published:** 2019-05-03

**Authors:** Guanghua Chen, Peng Li, Zhijun Liu, Rong Zeng, Xiaotang Ma, Yanfang Chen, Haijia Xu, Zhanghua Li, Hao Lin

**Affiliations:** a Department of Orthopedics, The First Clinical Medical College, Jinan University, Guangzhou, China; b Department of Orthopedics, Affiliated Hospital of Guangdong Medical University, Zhanjiang, China; c Department of Surgery, Guangdong Key Laboratory of Age-Related Cardiac and Cerebral Diseases, Affiliated Hospital of Guangdong Medical University, Zhanjiang, China; d Department of Pharmacology & Toxicology, Boonshoft School of Medicine, Wright State University, Dayton, OH, USA; e Department of Orthopedics, Tongren Hospital of Wuhan University, Wuhan, China

**Keywords:** Endothelial progenitor cells, microvesicles, miR-126, osteoblasts

## Abstract

**Objective**: To evaluate whether EPC-MVs could promote bone regeneration by directly regulating osteoblast through miR-126. The underlying mechanisms were also explored. **Methods**: EPCs were isolated from bone marrow mononuclear cells. EPC-MVs were collected from EPCs cultured medium. The lentivirus was used to induce miR-126 over-expression in EPCs and EPC-MVs. miR-126 expression was detected by qRT-PCR. The proliferation, migration, apoptosis and differentiation abilities of osteoblast cells MC3T3-E1 were analysed in the presence or absence of EPC-MVs or miR-126 overexpressed EPC-MVs (EPC-MVs-miR126). The proteins of Erk1/2 and Bcl-2 were analysed by western blot. Erk1/2 inhibitor was used for pathway exploration. **Results**: EPC-MVs reduced apoptosis and promoted proliferation and migration of MC3T3-E1 cells, which could be enhanced by miR-126 enrichment (*p*< 0.05). Neither EPC-MVs nor EPC-MVs-miR126 had an effect on MC3T3-E1 cell osteogenic differentiation (*p*> 0.05). EPC-MVs-miR126 had better effects than EPC-MVs on upregulating the expressions of p-Erk1/2 and Bcl-2, which were abolished by Erk1/2 inhibitor. ERK1/2-Bcl-2 activity plays a crucial role in the regulation of EPC-MVs/EPC-MVs-miR126 on the effect of MC3T3-E1 cells. **Conclusion**: EPC-MVs promote proliferation and migration of MC3T3-E1 cell while reduced apoptosis via the miR-126/Erk1/2-Bcl-2 pathway. A combination of EPC-MVs and miR-126 might provide novel therapeutic targets for bone regeneration and fracture healing through regulating osteoblast.

## Introduction

Fracture repair and bone regeneration has to go through a series of cellular events including inflammation, chondrogenesis, intramembranous and endochondral ossification ^[1].^ Among them, osteoblast proliferation is considered to be a major response at the beginning of fracture healing events [2]. It is well documented that bone formation largely relied on the prevention of osteoblast apoptosis [3]. Our previous study revealed that glucocorticoid-induced apoptosis in osteoblastic cells (MC3T3-E1) could be inhibited to improve osteoporosis [4], which suggest that promote osteoblast proliferation and inhibit apoptosis are important strategies for promoting bone repair. Furthermore, fracture healing is a complex process that involves both endochondral ossification, whereby bone formation occurs through a cartilage intermediate and intramembranous ossification, in which bone forms directly from differentiated osteoblasts. Thus, osteoblast differentiation is also important for bone regeneration [5].

Endothelial progenitor cells (EPCs) are circulating bone marrow-derived precursors, participating in tissue damage repair [6]. A recent study demonstrated that the therapeutic effects of EPCs are largely related to their released microvesicles (MVs) [7]. MVs are vesicles released when cells undergo activation or apoptosis, which can modulate cell functions through transferring their contents including proteins, mRNAs and miroRNAs (miRs) from mother cells to recipient cells, regulating recipient cell morphological and functional recovery [8]. A recent study showed that EPC-MVs could travel to the injured tissue, merge with the target cell and promote bone healing through stimulating neoangiogenesis [9]. In the process of bone repair, osteoblasts play a critical role in the direct regulation of ossification [10]. However, it is poorly understood whether EPC-MVs could promote bone regeneration by directly regulating osteoblast.

MiR-126 has been reported to promote EPC proliferation, migration, and inhibit apoptosis [11]. Our previous study has demonstrated that miR-126 over-expressed MVs from non-obese adipose tissue stem cell were able to induce endothelial cells migration and tube-like structure formation. Meanwhile, the effects of EPC-MVs are eliminated in diabetes due to the reduction of their carried miR-126 [12], which indicate that miR-126 is associated with the effects of EPC-MVs. However, whether miR-126 could promote the effects of EPC-MVs on osteoblast needs further investigation.

The Erk1/2-Bcl-2 signal pathway involves a series of vital cellular processes, such as proliferation, migration and apoptosis [13]. Activation of the Erk1/2-Bcl-2 pathway promotes pancreatic tumour cells survival and inhibits apoptosis [14]. Erk phosphorylation was reported to increase the survival of human macrophages, which was associated with Bcl-2 up-regulation [15]. All of these findings suggest that Erk1/2-Bcl-2 signalling pathway plays an important role in regulating cell survival and may participate in the regulation of osteoblast.

Therefore, the current study was designed to evaluate the effects of EPC-MVs on osteoblast cells MC3T3-E1 proliferation, migration, apoptosis and differentiation. Moreover, whether the underlying mechanisms were associated with miR-126/Erk1/2-Bcl-2 signalling pathways was also investigated.

## Material and methods

### EPCs isolation, culture and characterization

EPCs were isolated from bone marrow (BM) mononuclear cells (MNCs) by density centrifugation and characterized as previously described [16]. Briefly, male adult C57BL/6 mice (8–10 weeks, 25–30 g) were sacrificed and BM was flushed out from tibias and femurs. 1 × 10^7^ MNCs were plated on fibronectin-coated 6-well plates, then cultured in endothelial cell basal medium-2 (EBM-2) supplemented with 5% FBS containing EPC growth cytokine cocktail (Lonza, Walkersville, MD, USA). After 3 days, non-adherent cells were removed. Thereafter, the culture medium was changed every 2 days. Cultured cells were verified by Di-LDL and Bs-Lectin double staining.

### Overexpression of miR-126 in EPCs

The lentivirus containing murine miR-126 (Lenti-miR126) and lentivirus containing green fluorescence protein (Lenti-GFP) were obtained from GenePharma (GenePharma Co., Ltd., Shanghai, China). EPCs were seeded in 6-well plates (1 × 10^5^/well) for a confluence of 70%; then, lentivirus was added (at 5 × 10^6^ infection-forming units) for 6 h, after which the serum-free medium was changed by 10% (v/v) FBS medium for 72 h. The level of miR-126 in EPCs was analysed by real-time PCR (RT-PCR). The miR-126 enriched EPCs (EPC-miR126) were harvested for subsequent experiments.

### EPC-MVs isolation and characterization

EPC-MVs were collected from EPCs cultured medium as previously described [17]. In brief, EPCs cultured medium was collected and centrifuged at 300 g, 15 min, followed by 2000 g, 20 min to remove cell and debris. Cell-free supernatants were ultra-centrifuged at 20,000 g, 2 h to pellet MVs and resuspended in PBS. The number of MVs was counted by the nanoparticle tracking analysis system (NTA300, Malvern, Britain). The shape and morphology of MVs were detected by a JEM-1400 transmission electron microscope (TEM; JEOL, Tokyo, Japan) at an accelerating voltage of 80kV. Meanwhile, MVs were quantified by flow cytometry based on EPC related surface markers CD34 and VEGFR2 (1:300; BD Bioscience, San Jose, CA, USA) as previously reported [17]. MVs from EPC-miR126 (EPC-MVs-miR126) were collected and miR-126 expression was detected by qRT-PCR.

### Concentration–response study of EPC-MVs on MC3T3-E1 cell viability

To determine the dose-effect of EPC-MVs on MC3T3-E1 cell viability, cells were treated with different doses (0, 10^5^, 10^6^ or 10^7^ mL^−1^) of EPC-MVs. After 24 h and 48 h of co-culture, the cell viability was assessed using the CCK8 solution (Beyotime, Shanghai, China). The absorbance at 450 nm was detected using the spectrophotometer (Multiskan GO, Thermo scientific, Hudson, NH, USA).

### Cell proliferation assay

MC3T3-E1 cells were seeded at a concentration of 5,000 cells/well into 96-well plates and grown at 37℃ for 24 h. For pathway blocking experiments, cells were pre-incubated with Erk1/2 inhibitor PD98059 (10 μM) for 2 h. Then, cells were treated with EPC-MVs or EPC-MVs-miR126 (10^6^ mL^−1^). Cell proliferation was detected at 24 h and 48 h after treatment by using CCK8 solution.

### Wound healing assay

The wound healing assay protocol has been described in our previous study [18]. Briefly, MC3T3-E1 cells were seeded at a concentration of 2 × 10^5^/well into 6-well plates. The monolayers were scratched using a 200 μL sterile micropipette tip when the cells reached confluence. After removing floating cells, cells were then cultured in fresh serum-free medium and treated with EPC-MVs or EPC-MVs-miR126 for 48 h. The scratch was observed under the microscope. For pathway blocking experiments, cells were pre-incubated with PD98059 (10 μM) for 2 h. The cell migration rate was counted in four different vision fields.

### Cell apoptosis assay

Cell apoptosis assay was assessed by using PE Annexin V Apoptosis Detection Kit I (BD Biosciences, San Diego, CA, USA) and Hoechst 33258 staining as previously described [18]. Briefly, following different treatments, MC3T3-E1 cells were collected and resuspended in 100 μL binding buffer, stained with Annexin V-PE and 7-AAD at room temperature in the dark for 15 min. Stained cells were analysed using a FACSCantoⅡ flow cytometer (BD Biosciences, USA). For pathway blocking experiments, cells were pre-incubated with PD98059 (10 μM) for 2 h, then cells were treated with EPC-MVs or EPC-MVs-miR126. Cell apoptosis rate was quantified in three times independent experiments.

For Hoechst 33258 staining, cells were washed with PBS and fixed with 4% paraformaldehyde for 10 min after EPC-MVs or EPC-MVs-miR126 treatment for 48 h, then stained with Hoechst 33258 (Sigma, Louis, MO, USA) according to the manufacturer’s instruction. Morphologic changes were observed under a fluorescence microscopy in four independent fields.

### Gene expression analysis

The levels of miR-126 in EPCs and EPC-MVs were analysed. Briefly, total RNA was extracted by using miRcute miRNA isolation kit according to the manufacturer’s instructions. cDNA was synthesized using miRcute miRNA First-Strand cDNA Synthesis kit. RT-PCR was conducted with miR-126 specific primers and miRcute miRNA qPCR Detection Kit (SYBR Green) (Tiangen, Beijing, China). Small nuclear RNA U6 was used as an internal control. Real-time PCR was performed with a LightCycler® 480ⅡReal-Time thermal cycler (Roche, Basel, Swiss). The cycling condition was carried out as follows: 95°C for 30 s, followed by 40 cycles of 95°C for 5 s and 60°C for 20 s. The relative quantification of gene expression was presented with the values of 2^–ΔΔCT^ method.

### Osteogenic differentiation and mineralization assay

Osteogenic differentiation of MC3T3-E1 cells was induced by osteogenic induction medium (OIM) containing 10% FBS, 10^–7^ M dexamethasone, 10 mM β-glycerophosphate and 50 µM ascorbate-2-phosphate (Sigma, USA). The OIM and MVs were changed every 3 days. Following the induction with OIM for 21 days, mineralization assay was performed to evaluate calcium deposit formation. Cells were fixed with 70% ethanol for 15 min, then stained with 0.5% Alizarin red S (pH 4.1) for 10 min and washed three times with distilled water. The phase contrast images were captured using an inverted microscope. For quantification of the deposited Alizarin Red S, the dye was extracted using 10% cetylpyridinium chloride solution (CPC) for 1 h at room temperature and absorbance was measured at 550 nm.

### RT-PCR detection of osteogenesis expression

MC3T3-E1 cells were treated with or without EPC-MVs or EPC-MVs-miR126 in OIM condition for 14 days. Total RNA was isolated as described above. Total RNA (1 μg) was reverse-transcribed into cDNA using PrimeScript RT Enzyme mix (Takara Bio Inc., Shiga, Japan) at 37°C for 15 min, followed by an 85°C incubation for 5 s. Osteogenic-specific primers such as Runx2, Osteopontin (OPN) and Osteocalcin (OCN) were detected. GAPDH was used as an internal control. Fold change in gene expression was calculated based on the normalized mean differences method.

### Western blot

The treated cells were collected and lysed with cell lysis buffer, and then the protein concentrations were determined using the BCA Protein Assay Kit (Thermo Scientific, Waltham, MA, USA). Total cell protein (20 μg) was separated by 10% sodium dodecyl sulfate-polyacrylamide gel electrophoresis (SDS-PAGE) and transferred to PVDF membranes (Millipore Corp., Billerica, MA, USA). The membranes were then blocked for 1 h at RT in 5% non-fat milk and washed in TBST (0.5% Tween 20 in TBS). Primary antibodies were added overnight at 4℃, and then membranes were washed with TBST and incubated for 1 h with secondary antibodies. The antibody-reactive bands were detected using Pierce™ ECL Western Blotting Substrate (Thermo Scientific) and exposed to radiographic film.

### Statistical analysis

All data are presented as the mean ± standard deviation (SD). Statistical significance of the data was evaluated by either a Student’s *t*‑test or one-way ANOVA. *P*< 0.05 was considered to indicate a statistically significant difference.

## Results

### Characterization of EPCs and EPC-MVs

**Figure 1.**
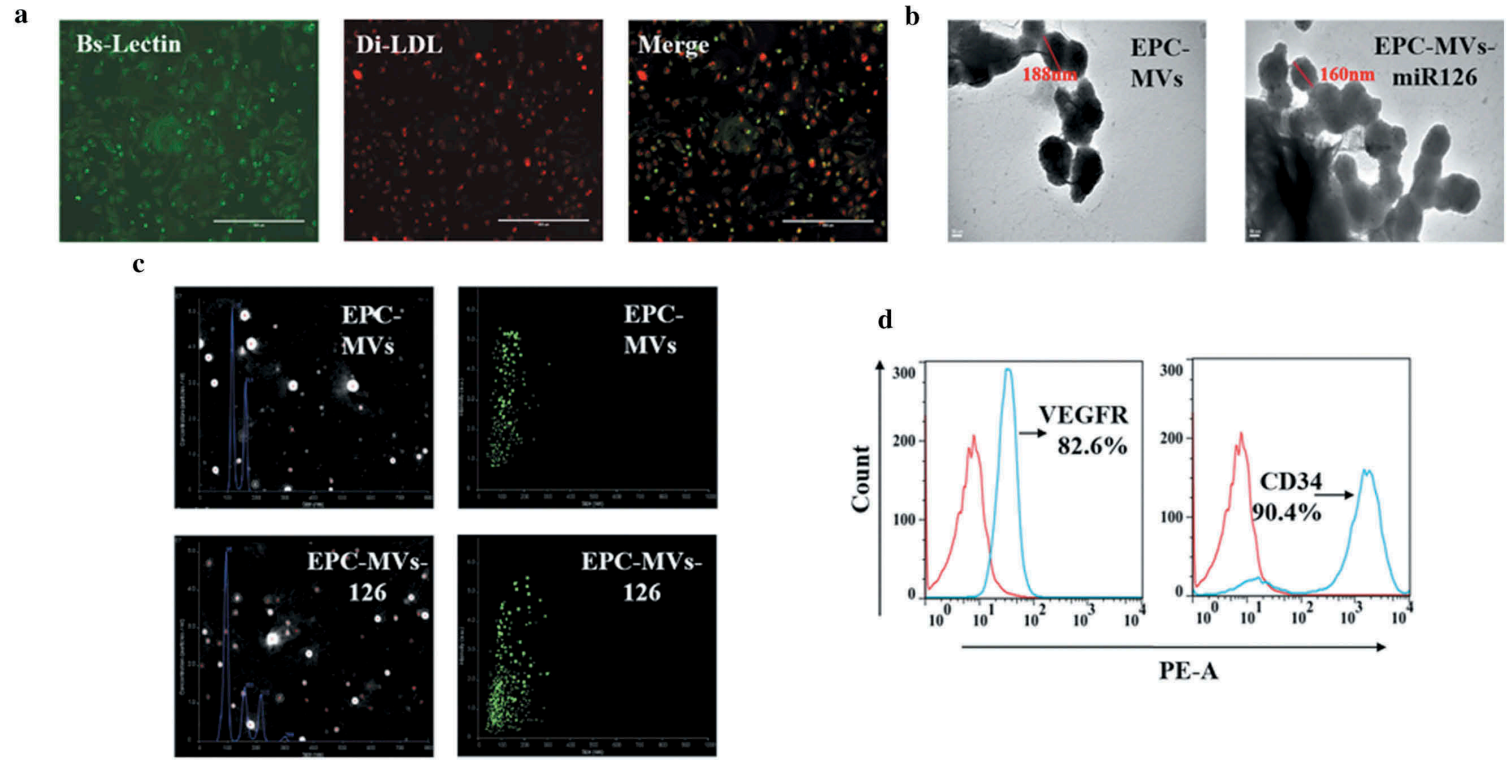
Characterization of EPCs and EPC-MVs. (a) EPCs were identified by Di-LDL and Bs-Lectin staining. (b) Representative image of EPC-MVs and EPC-MVs-miR126 examined by TEM. (c) Size distribution of EPC-MVs and EPC-MVs-miR126 detected by NTA. (d) EPC-MVs stained with PE-CD34 and PE-VEGFR were analysed by flow cytometry.

EPCs were defined as double-positive of Di-LDL and Bs-Lectin staining (Figure 1(a)). Transmission electron microscopy results revealed that EPC-MVs and EPC-MVs-miR126 have similar spheroid morphology, and their diameter size was approximately 188 nm and 160 nm, respectively (Figure 1(b)). According to NTA analysis, the concentration of MVs was 10^9^/mL, and there was no difference of the number between EPC-MVs and EPC-MVs-miR126 (*p*> 0.05) (Figure 1(c)), suggesting that miR-126 transfection did not change the release of MVs. Flow cytometry showed that both EPC-MVs and EPC-MVs-miR126 positively expressed EPC specific markers (CD34 and VEGFR2) (Figure 1(d)).

### Lenti-miR126 transduction increased miR-126 expression in EPCs and EPC-MVs

**Figure 2.**
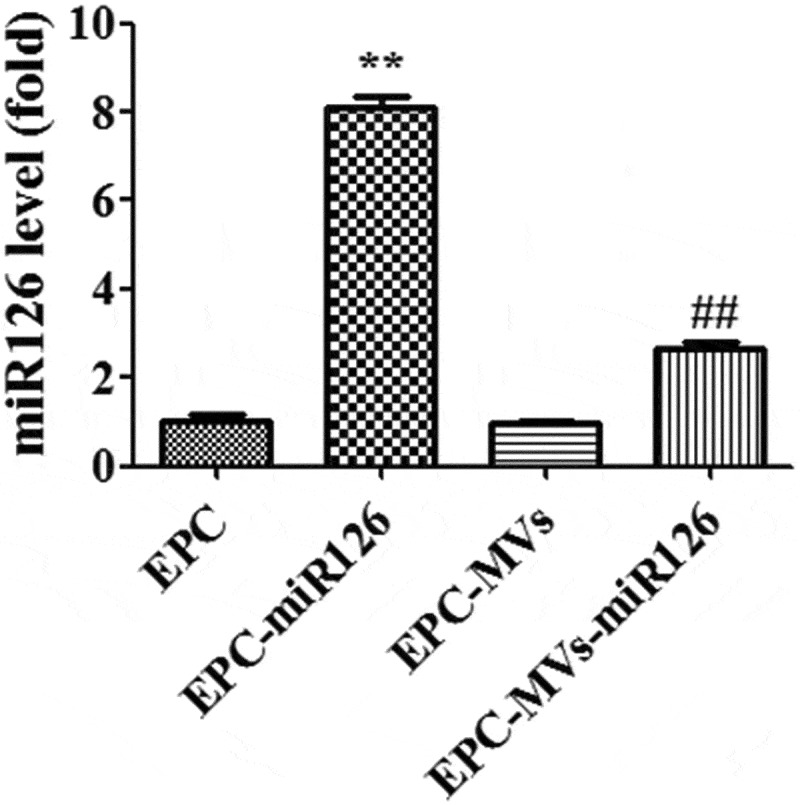
The expression of miR126 in different groups. **p< 0.01 vs. EPC, ^##^*p*< 0.01 vs. EPC-MVs.

RT-PCR results showed that lenti-miR126 transfection increased the miR126 level in EPC-miR126 to 8.2-fold of EPCs. Moreover, miR-126 was up-regulated in EPC-MVs-miR126 compared with that in EPC-MVs (*p*< 0.05) (Figure 2).

### EPC-MVs-miR126 had better effects than EPC-MVs on promoting MC3T3-E1 cells proliferation

**Figure 3.**
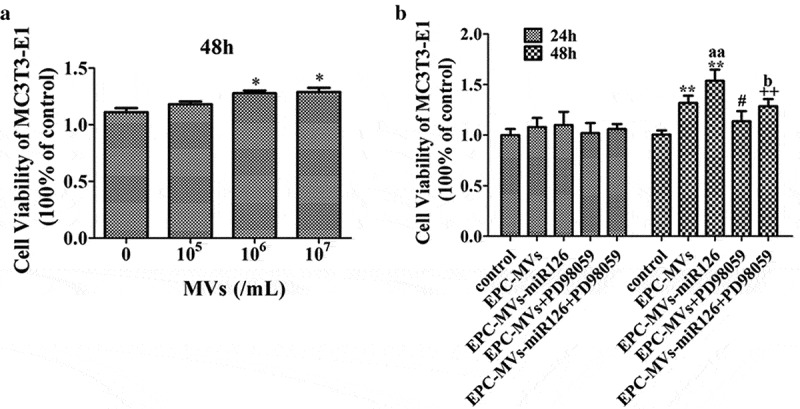
The effects of EPC-MVs and EPC-MVs-miR126 on the proliferation of MC3T3-E1 cells. (a) The effects of different concentrations of MVs on MC3T3-E1 cell viability at 48 h. (b) The proliferation of MC3T3-E1 cell in different groups. *p< 0.05, **p< 0.01 vs. control; ^#^*p*< 0.05 vs. EPC-MVs; ^++^*p*< 0.01 vs. EPC-MVs-miR126; ^aa^*p*<0.01 vs. EPC-MVs; ^b^*p*<0.05 vs. EPC-MVs+PD98059.

In the dose-effect experiments, determining the effects of MVs on cell proliferation by treating the MC3T3-E1 cells with different concentrations of MVs for 24 h and 48 h, it showed that MVs treatment for 24 h did not change the proliferation ability of MC3T3-E1 cells (data not shown). The viability of cells treated with EPC-MVs (10^5^/mL, 10^6^/mL, 10^7^/mL) for 48 h was 106%, 115% and 117%, respectively (Figure 3(a)). These data reflect that with the increase of the MVs concentration, the number of viable cells was also increased. Therefore, 10^6^/mL EPC-MVs were selected for the following experiments.

EPC-MVs significantly promoted MC3T3-E1 cell proliferation after 48 h treatment, which was further enhanced by EPC-MVs-miR126 (*p*< 0.05) (Figure 3(b)). However, pre-incubation of MC3T3-E1 cells with PD98059 inhibited this effect of EPC-MVs/EPC-MVs-miR126.

### EPC-MVs/EPC-MVs-miR126 enhanced the migration ability of MC3T3-E1 cells

**Figure 4.**
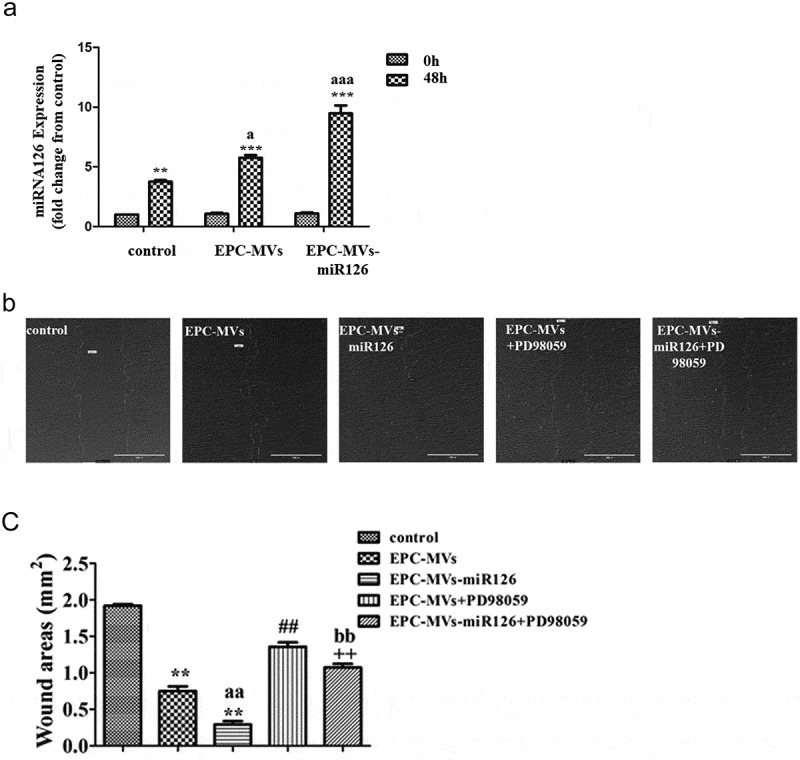
The effects of EPC-MVs and EPC-MVs-miR126 on the migration of MC3T3-E1 cells. (a) The expression of miR126 before and after wounding. (b) Representative image of cell migration at 48 h of MVs treatment. (c) Summarized data on cell migration. **p< 0.01 vs. control; ^##^*p*< 0.01 vs. EPC-MVs; ^++^*p*< 0.01 vs. EPC-MVs-miR126; ^aa^*p*<0.01 vs. EPC-MVs; ^bb^*p*<0.01 vs. EPC-MVs+PD98059.

The expression of miR126 before and after wounding was measured by RT-PCR. After wounding, the expression of miR126 was significantly increased in control, EPC-MVs and EPC-MVs-miR126-treated cells (*p*< 0.05) (Figure 4(a)).

The covered wound areas of EPC-MVs-treated cells were significantly increased compared with the control after 48 h treatment (*p*< 0.05), suggesting the migration ability of MC3T3-E1 was enhanced after EPC-MVs incubation. Interestingly, it was found that the migration ability of EPC-MVs-miR126-treated cells was higher than that of EPC-MVs-treated cells, indicating that miR-126 contributed to the effects of MVs on MC3T3-E1 migration. Pre-incubation with PD98059 attenuated the effects of EPC-MVs/EPC-MVs-miR126 on promoting MC3T3-E1 cell migration (Figure 4(b,c)).

### Anti-apoptotic effect of EPC-MVs on MC3T3-E1 cells promoted by EPC-MVs/EPC-MVs-miR126

**Figure 5.**
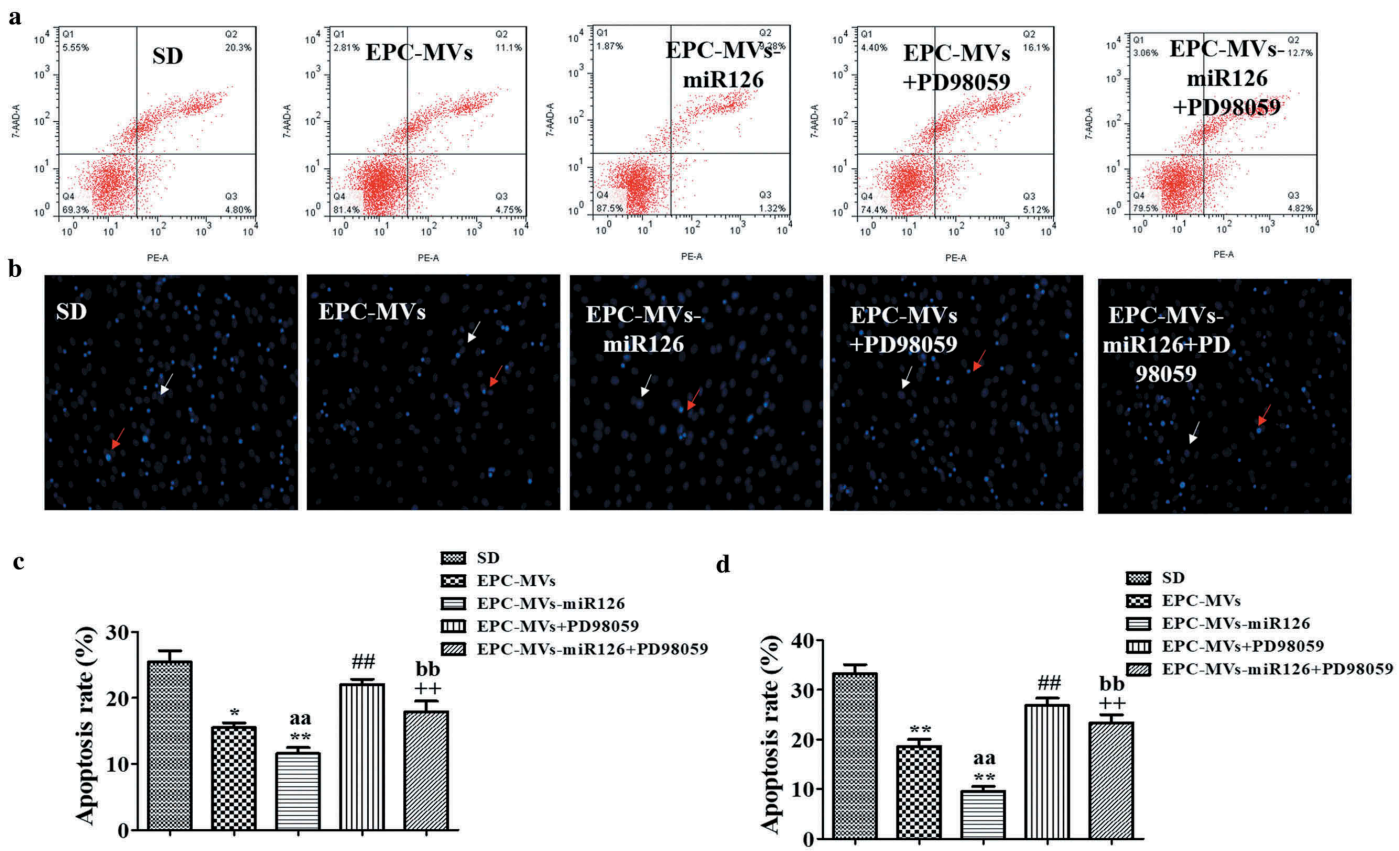
The effects of EPC-MVs and EPC-MVs-miR126 on apoptosis of MC3T3-E1 cell. (a) Cell apoptosis was measured by flow cytometry. (b) Cell apoptosis was determined by Hoechst 33258 staining (Red arrows represent apoptosis cells, white arrows represent normal cells). (c) and (d) Summarized data on apoptosis rate (%) assessed by flow cytometry and Hoechst 33258 staining. *p< 0.05, **p< 0.01 vs. SD; ^##^*p*< 0.01 vs. EPC-MVs; ^++^*p*< 0.01 vs. EPC-MVs-miR126; ^aa^*p*<0.01 vs. EPC-MVs; ^bb^*p*<0.01 vs. EPC-MVs+PD98059.

EPC-MVs significantly decreased the apoptotic rate of MC3T3-E1 (25.7 ± 2.45% in SD group, 15.49 ± 2.34% in EPC-MVs group). Moreover, EPC-MVs-miR126 exhibited better anti-apoptotic effect than that of EPC-MVs (9.37 ± 0.66% in EPC-MVs-miR126 group) (Figure 5(a,c)).

Hoechst 33258 staining also revealed that MVs significantly reduced the apoptotic rate of MC3T3-E1 (33.24 ± 1.82% in SD group, 18.57 ± 1.48% in EPC-MVs group, 9.56 ± 0.99% in EPC-MVs-miR126 group). Again, PD98059 eliminated the anti-apoptotic effect of EPC-MVs/EPC-MVs-miR126 (Figure 5(b,d)).

Altogether, these results showed that EPC-MVs-miR126 seemed to be more effective than EPC-MVs for preventing cell apoptosis.

### Erk1/2-Bcl2 pathway was involved in the effects of EPC-MVs/EPC-MVs-miR126 on MC3T3-E1 cells

**Figure 6.**
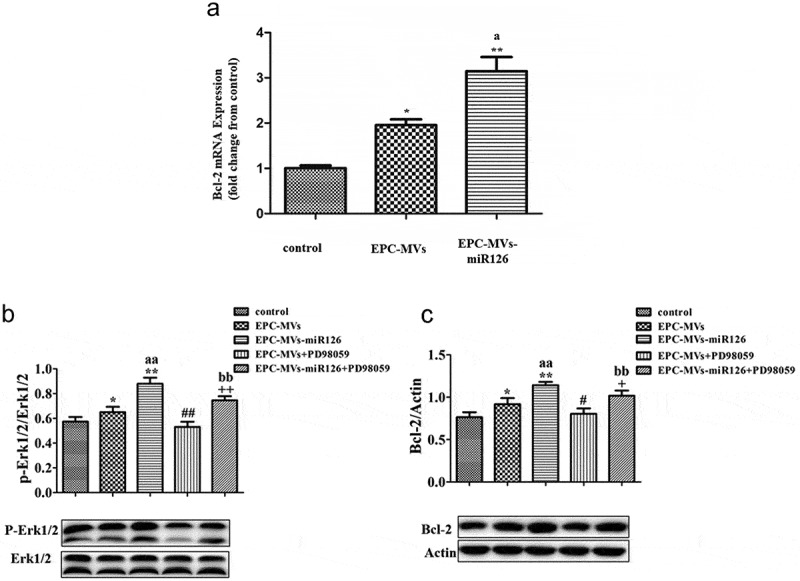
The effects of EPC-MVs and EPC-MVs-miR126 on Erk1/2-Bcl2 pathway. (a) EPC-MVs group and EPC-MVs-miR126 group increase the expression of Bcl-2 gene in MC3T3-E1 cell. (b) Expression of p-Erk1/2/Erk1/2. (c) Expression of Bcl-2. *p< 0.05 vs. control; ^##^*p*< 0.01 vs. EPC-MVs; ^+^*p*< 0.05, ^++^*p*< 0.01 vs. EPC-MVs-miR126; ^aa^*p*<0.01 vs. EPC-MVs; ^bb^*p*<0.01 vs. EPC-MVs+PD98059.

RT-PCR showed that EPC-MVs group and EPC-MVs-miR126 group significantly increase the expression of the Bcl-2 gene in MC3T3-E1 cell, compared with the control group (*p*< 0.05, Figure 6(a)). Compared with the control, EPC-MVs treatment significantly increased levels of Bcl-2 and p-Erk1/2 protein expression in MC3T3-E1 (vs. control; *p*< 0.05; n = 3/group; Figure 6(b,c)). Meanwhile, EPC-MVs-miR126 was more effective on the increase of Bcl-2 and p-Erk1/2 compared with EPC-MVs (*p*< 0.01; n = 3/group).

To further confirm the role of Erk1/2-Bcl-2 pathway, we applied Erk inhibitor PD98059. The expression of p-Erk1/2/Erk1/2 and Bcl-2 protein was inhibited by PD98059 (Figure 6(a,b)) (*p*< 0.05, *p*< 0.01; n = 3/group). As described above, PD98059 inhibited the effect of EPC-MVs/EPC-MVs-miR126 on cell proliferation, migration and apoptosis. Moreover, compared with EPC-MVs, the inhibition effects of PD98059 in EPC-MVs-miR126 on MC3T3-E1 cells functions were more obvious. In summary, these results suggest that ERK1/2-Bcl-2 activity plays a crucial role in the regulation of EPC-MVs/EPC-MVs-miR126 on the effect of MC3T3-E1 cells.

### Effects of EPC-MVs/EPC-MVs-miR126 on the differentiation of MC3T3-E1 cells

**Figure 7.**
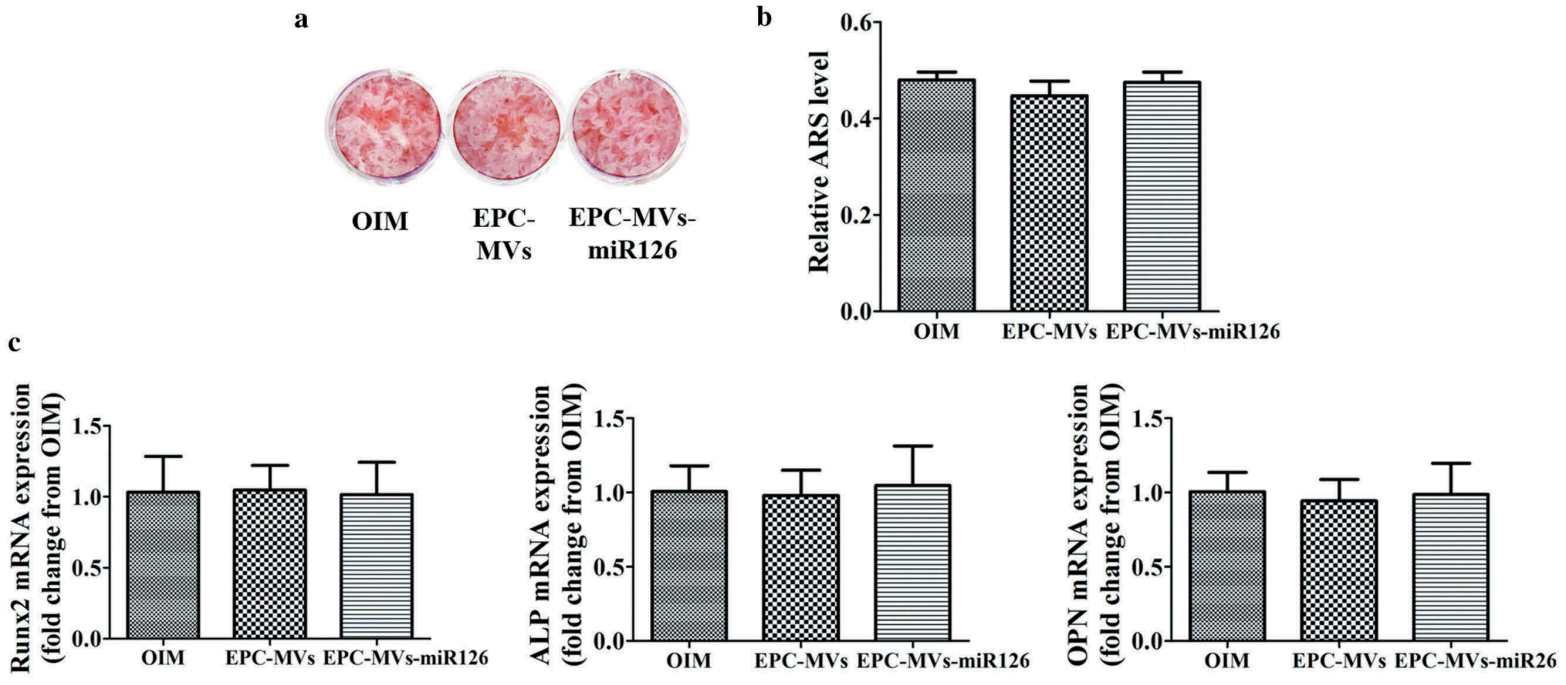
The effects of EPC-MVs and EPC-MVs-miR126 on osteogenic differentiation in MC3T3-E1 cell. (a) The mineralized nodules were stained by Alizarin Red S. (b) The mineralization was quantified with 10% CPC. (c) The mRNA expression of osteogenic genes (Runx2, ALP and OPN) at day 14.

To clarify the influence of EPC-MVs/EPC-MVs-miR126 on the differentiation of MC3T3-E1 cells, Alizarin Red S staining and quantitation were performed at day 21d. The results showed that both EPC-MVs and EPC-MVs-miR126 had no effects on MC3T3-E1 cells osteogenesis differentiation compared with the positive control (*p*< 0.05) (Figure 7(a,b)). Meanwhile, RT-PCR results showed that the levels of osteogenic-specific gene mRNA (ALP, Runx2 and OPN) were not significantly affected (*p*< 0.05) (Figure 7(c)).

### Effects of knock-down of miR126 on MC3T3-E1 cells

**Figure 8.**
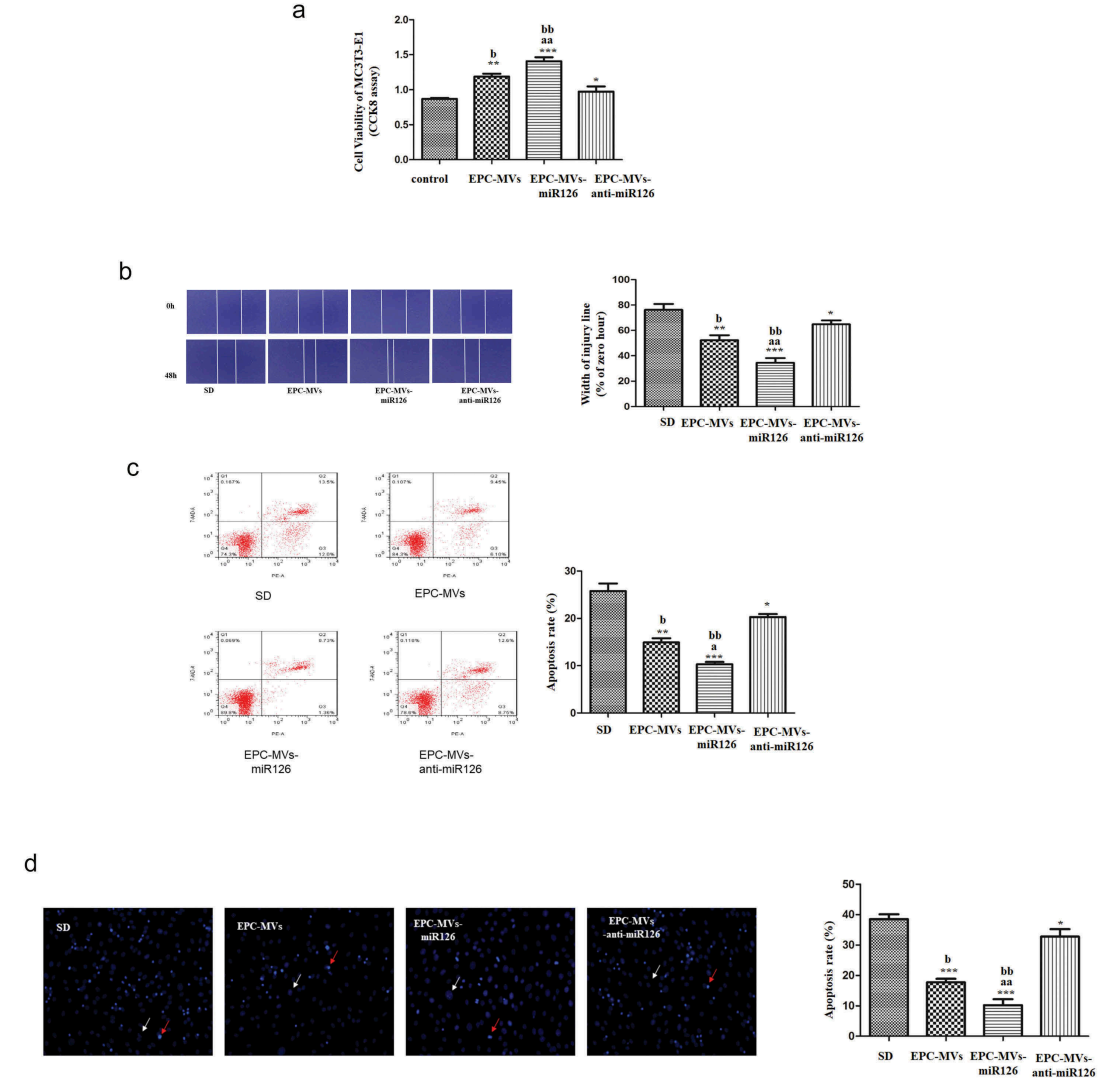
Effects of knock-down of miR126 on MC3T3-E1 cells. (a) The proliferation of MC3T3-E1 cell in different groups. (b) Representative image of cell migration and summarized data on cell migration. (c) Cell apoptosis was measured by flow cytometry. (d) Summarized data on apoptosis rate (%) assessed Hoechst 33258 staining. *p< 0.05, **p< 0.01 vs. SD; ^##^*p*< 0.01 vs. EPC-MVs; ^++^*p*< 0.01 vs. EPC-MVs-miR126; ^aa^*p*<0.01 vs. EPC-MVs; ^bb^*p*<0.01 vs. EPC-MVs-anti-miR126.

Knock-down of miR126 significantly reduced MC3T3-E1 cell proliferation compared with EPC-MVs-miR126 (p < 0.05) (Figure 8(a)). Moreover, knock-down of miR126 attenuated the effects of EPC-MVs/EPC-MVs-miR126 on promoting MC3T3-E1 cell migration (p < 0.05) (Figure 8(b)). In the apoptosis analysis, the knock-down of miR126 showed similar results. Knock-down of miR126 attenuated the effects of EPC-MVs/EPC-MVs-miR126 on promoting MC3T3-E1 cell apoptosis (p < 0.05) (Figure 8(c,d)). After the knock-down of miR126, the cells proliferation, migration and apoptosis were still affected compared with the control group, which suggested that, besides miR126, there should be other components that still play roles in osteoblasts.

## Discussion

In the current study, the effects of EPC-MVs on osteoblast cells were identified. EPC-MVs could promote MC3T3-E1 cell proliferation and migration while inhibiting cell apoptosis. Moreover, EPC-MVs overexpressing miR-126 was more effective. Meanwhile, the effects of MVs on MC3T3-E1 cell function were associated with Erk1/2-Bcl-2 signalling pathway. However, MVs have no influence on MC3T3-E1 cell osteogenic differentiation. It suggested that EPC-MVs promoted proliferation and migration of MC3T3-E1 cells and reduced apoptosis via Erk1/2-Bcl-2 pathway. MiR-126 could further enhance the effects of EPC-MVs on MC3T3-E1 cells.

Osteoblasts are key components for bone formation. It was demonstrated that MVs derived from mesenchymal stem cell stimulate bone regeneration by directly regulating osteoblast proliferation [19]. In this study, we showed that EPC-MVs promoted MC3T3-E1 cells proliferation at a concentration of 10^6^/mL, reflecting that EPC-MVs might stimulate bone regeneration. A previous study has revealed that 10 μg/ml EPC-MVs exhibited a proliferative effect on ECs [20]. The concentration discrepancy of EPC-MVs may be due to the difference in cell types.

EPC-MVs have been reported to promote endothelial cell survival, proliferation and tube formation ability via their carried miR-126 [20]. Our previous report has also shown that miR-126 enriched EPC-MVs ameliorated H/R induced human brain microvascular endothelial cell apoptosis and dysfunction. All these results indicate that EPC-MVs rich in miR-126 may exert beneficial effects on recipient cells. In the present study, EPC-MVs-miR126 also showed a stronger effect on the promotion of MC3T3-E1 cell viability compared with EPC-MVs in a time-dependent manner. Furthermore, the role of EPC-MVs-miR126 in accelerating cell migration was more obvious than EPC-MVs treatment, which was consistent with the results of cell viability. It demonstrated that miR-126 could enhance the effects of EPC-MVs on increasing osteoblast proliferation and migration.

Osteoblast apoptosis plays a critical role in skeletal maturation and bone turnover during modelling and remodelling processes [21]. Recently, it has highlighted a link between osteogenic cell death and osteoporosis. Prevention of osteoporosis can be achieved by inhibiting osteoblast and osteocyte apoptosis [22]. Previous studies have demonstrated that EPC-MVs inhibited apoptosis of several cells, such as renal cells and brain vascular smooth cells [6,18]. Here, our data showed that EPC-MVs could inhibit apoptosis of MC3T3-E1 cells, indicating that EPC-MVs might promote bone formation through inhibiting osteoblast apoptosis. Interestingly, EPC-MVs-miR126 showed again stronger effect on inhibiting cell apoptosis.

Erk1/2 pathway participates in the regulation of osteoblast proliferation and migration, and Erk1/2 phosphorylation is required for the stabilization of Bcl-2 family during osteoblast apoptosis [13]. To confirm the involvement of the Erk1/2-Bcl2 pathway, whether EPC-MVs/EPC-MVs-miR126 activates Erk1/2 in MC3T3-E1 cell was examined. The effects of EPC-MVs/EPC-MVs-miR126 on cell proliferation, migration and apoptosis were blocked by Erk1/2 inhibitor PD98059, indicating that EPC-MVs/EPC-MVs-miR126 regulated these activities of MC3T3-E1 via Erk1/2-Bcl-2 pathway. Meanwhile, when the Erk1/2 pathway was blocked, the effects of EPC-MVs-miR126 were more slightly inhibited than that of EPC-MVs, indicating that there should be other signal pathways than Erk1/2 pathway involving in the miR-126 promoted the effects of EPC-MVs.

Osteoblast differentiation is regulated by various hormones including PTH, estrogen, glucocorticoids and local factors in a paracrine and/or an autocrine fashion [23]. Previous study has shown that MVs released from hormone-refractory prostate cancer cells facilitate MC3T3-E1 cells differentiation [24]. However, in this study, there was no significant increase in the expression of osteogenesis-related genes (ALP, Runx2 and OPN) after the treatment of OIM and EPC-MVs/EPC-MVs-miR126 in MC3T3-E1 cells. Furthermore, Alizarin Red S staining confirmed that EPC-MVs/EPC-MVs-miR126 has no marked effects on increasing mineralization of MC3T3-E1 cells. The discrepancy may be caused by the difference in protein, mRNA and miRNA derived from different parent cells.

In conclusion, the current study revealed that EPC-MVs promoted proliferation and migration of MC3T3-E1 cell and reduced apoptosis via Erk1/2-Bcl-2 pathway. Meanwhile, miR-126 overexpression could further enhance the effect of EPC-MVs. A combination of EPC-MVs and miR-126 might provide novel therapeutic targets for bone regeneration and fracture healing through regulating osteoblast.
