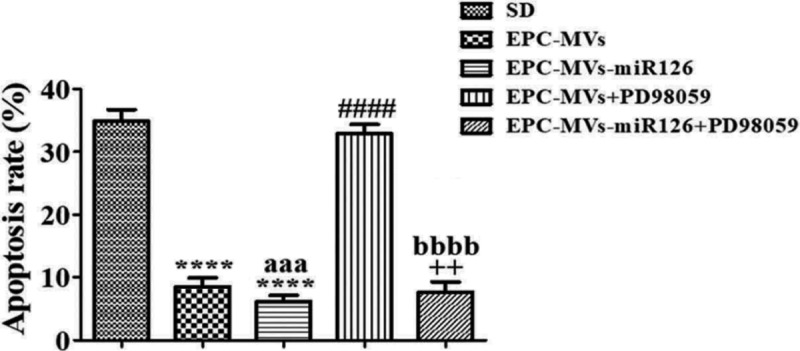# Correction

**DOI:** 10.1080/19336896.2026.2684820

**Published:** 2026-06-10

**Article title**: Enrichment of miR-126 enhances the effects of endothelial progenitor cell–derived microvesicles on modulating MC3T3-E1 cell function via Erk1/2-Bcl-2 signalling pathway

**Authors**: Chen, G., Li, P., Liu, Z., Zeng, R., Ma, X., Chen, Y., Xu, H., Li, Z., & Lin, H.

**Journal**: *Prion*

**Bibliometrics**: Volume 13, Number 01, pages 106 - 115

**DOI**: https://doi.org/10.1080/19336896.2019.1607464

The author has reported that Figure 5a and 5c appear incorrect in the published article and has requested that they be updated to display correctly.

The author has requested that the current versions of Figure 5a and 5c be replaced with the updated versions provided below, as these more accurately reflect the original intention.

**Figure 5a**

**Figure:**
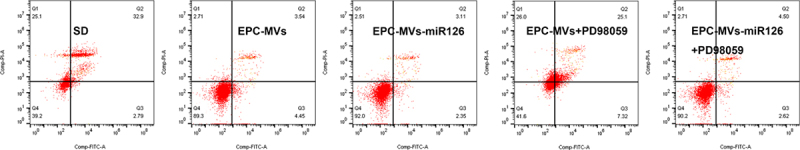

**Figure 5c**

**Figure:**